# Antimicrobial resistance among pathogenic bacteria from mink (*Neovison vison*) in Denmark

**DOI:** 10.1186/s13028-017-0328-6

**Published:** 2017-09-13

**Authors:** Nanett Kvist Nikolaisen, Desireé Corvera Kløve Lassen, Mariann Chriél, Gitte Larsen, Vibeke Frøkjær Jensen, Karl Pedersen

**Affiliations:** National Veterinary Institute, Technical University of Denmark, Kemitorvet, Anker Engelundsvej 1, 2800 Lyngby, Denmark

**Keywords:** Antimicrobial consumption, Antimicrobial resistance, *Escherichia coli*, Mink, *Neovison vison*, *Pseudomonas aeruginosa*, *Staphylococcus delphini*, *Streptococcus canis*

## Abstract

**Background:**

For proper treatment of bacterial infections in mink, knowledge of the causative agents and their antimicrobial susceptibility patterns is crucial. The used antimicrobials are in general not registered for mink, i.e. most usage is “off-label”. In this study, we report the patterns of antimicrobial resistance among pathogenic bacteria isolated from Danish mink during the period 2014–2016. The aim of this investigation was to provide data on antimicrobial resistance and consumption, to serve as background knowledge for new veterinary guidelines for prudent and optimal antimicrobial usage in mink.

**Results:**

A total number of 308 *Escherichia coli* isolates, 41 *Pseudomonas aeruginosa*, 36 *Streptococcus canis*, 30 *Streptococcus dysgalactiae*, 55 *Staphylococcus delphini*, 9 *Staphylococcus aureus*, and 20 *Staphylococcus schleiferi* were included in this study. Among *E. coli*, resistance was observed more frequently among the hemolytic isolates than among the non-hemolytic ones. The highest frequency of resistance was found to ampicillin, 82.3% and 48.0% of the hemolytic of the non-hemolytic isolates, respectively. The majority of the *P. aeruginosa* isolates were only sensitive to ciprofloxacin and gentamicin. Among the *Staphylococcus* spp., the highest occurrence of resistance was found for tetracycline. Regarding the nine *S. aureus,* one isolate was resistant to cefoxitin indicating it was a methicillin-resistant *Staphylococcus aureus*. Both β-hemolytic *Streptococcus* species showed high levels of resistance to tetracycline and erythromycin. The antimicrobial consumption increased significantly during 2007–2012, and fluctuated at a high level during 2012–2016, except for a temporary drop in 2013–2014. The majority of the prescribed antimicrobials were aminopenicillins followed by tetracyclines and macrolides.

**Conclusions:**

The study showed that antimicrobial resistance was common in most pathogenic bacteria from mink, in particular hemolytic *E. coli*. There is a need of guidelines for prudent use of antimicrobials for mink.

**Electronic supplementary material:**

The online version of this article (doi:10.1186/s13028-017-0328-6) contains supplementary material, which is available to authorized users.

## Background

The Danish production of mink (*Neovison vison*) skins was over 17 million annually (2013–2016). In 2016, this corresponded to 30% of the world production of 55.7 million skins [1]. In the Danish mink production, a range of bacterial species are causing a wide variety of infectious diseases. Among the most important ones are *Escherichia coli* (causing e.g. enteritis, pneumonia, and septicemia), *Streptococcus canis* and *Streptococcus dysgalactiae* (e.g. pneumonia, wound infections, and mastitis), various staphylococci such as *Staphylococcus delphini*, *Staphylococcus aureus*, and *Staphylococcus schleiferi* (e.g. wound infections, dermatitis, pleuritis, pneumonia, and mastitis) and *Pseudomonas aeruginosa* (e.g. hemorrhagic pneumonia) [2]. Antimicrobials are prescribed for treatment of these infections, but the usage of antimicrobial drugs may lead to the selection for resistance [3, 4]. Therefore, it is important to follow the development of resistance over time for the major bacterial pathogens. The consumption of antimicrobials for mink in Denmark increased over several years up to 2012 [5, 6]. Rising public focus on animal welfare may have contributed to the increase in 2011–2012 [6]. On the other hand, rising focus on antimicrobial consumption in the mink production may have contributed to the significant decrease in 2013 and 2014 [5, 6].

At present, only one antimicrobial product containing oxytetracycline is registered specifically for use in mink on the Danish market. Therefore, most antimicrobial use is “off-label” and dosages are extrapolated from other animal species, for which the products are registered, while knowledge on absorption and plasma concentrations in mink are sparse.

Here we present the results of the surveillance of antimicrobial resistance among pathogenic bacteria isolated from mink submitted for diagnostic at the National Veterinary Laboratory in a 3-year period, 2014–2016, and compare the results with previous data. The reported findings of antimicrobial resistance levels are discussed in relation to patterns in antimicrobial prescription for mink.

## Methods

### Bacterial isolates and culture conditions

Bacterial isolates were obtained from clinical samples from carcasses submitted to the National Veterinary Institute, DTU, during the period 2014–2016. The isolates were considered causative agents in infections that had led to the submission of the animals for laboratory examination. They had been recovered from pathological material by conventional culture methods and identified by matrix-associated laser desorption/ionization—time of flight mass spectrometry (MALDI-TOF MS). Mass spectra were obtained using an Autoflex Speed instrument (Bruker Daltonics, Bremen, Germany) calibrated with the Bruker *Escherichia coli* Bacterial Test Standard for Mass Spectrometry. Isolates were analysed with the MALDI Biotyper RTC 3.1 software using a BDAL database of library spectra (Bruker Daltonics). Only one isolate was included from each submission. They originated from many farms (n = 284 out of approx. 1400 Danish mink farms) and were assumed to be representative for Danish mink farms.

The *E. coli* isolates (n = 308) consisted of 158 hemolytic and 150 non-hemolytic isolates. They were derived from samples of liver, lung, mammary gland, feces, intestine, spleen, or uterus. The *S. canis* (n = 36) and *S. dysgalactiae* (n = 30) isolates were derived from mammary gland, liver, lung, paw, skin, or thoracic cavity. The staphylococci included in this investigation were primarily of the species *S. delphini* (n = 55) and a few of *S. aureus* (n = 9) or *S. schleiferi* (n = 20). They were derived from lung, liver, urine, skin, uterus, nose, or kidney. Isolates of *P. aeruginosa* (n = 41) were mainly isolated from the lung, except a few deriving from the spleen, liver, or thoracic cavity; all *P. aeruginosa* isolates were found in association with outbreaks of hemorrhagic pneumonia.

### Antimicrobial susceptibility testing

The minimal inhibitory concentration (MIC) of different antimicrobial agents was determined by the broth dilution susceptibility testing method using a semiautomatic system (SensiTitre, Trek Diagnostic Systems Ltd., UK) according to recommendations by the Clinical Laboratory Standards Institute [7]. The susceptibility test-panels and their test ranges are presented in Tables 1, 2, 3, 4, 5, 6 and 7. In the test result for *P. aeruginosa,* only apramycin, ciprofloxacin, colistin, gentamicin, spectinomycin, and streptomycin were reported due to intrinsic resistance towards the remaining antimicrobials [8, 9] (Table 3).

**Table 1:**
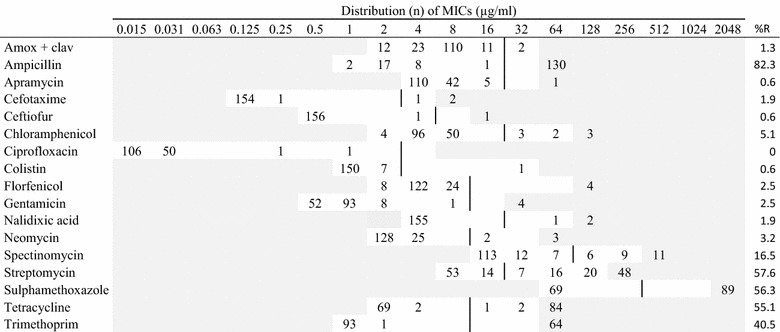
MIC distributions and occurrence of resistance of hemolytic *Escherichia coli* (n = 158) isolates from Danish mink (2014–2016)

Vertical lines indicate breakpoints for resistance (see breakpoint table in Additional file 1 A). White fields indicate test range for each antimicrobial. Values greater than the test range represent MIC values greater than the highest concentration in the range. MICs equal to or lower than the lowest concentration, are given as the lowest concentration in the test range

*R* resistance, *n* number of isolates, *amox* *+* *clav* amoxicillin with clavulanic acid (1:2)

**Table 2:**
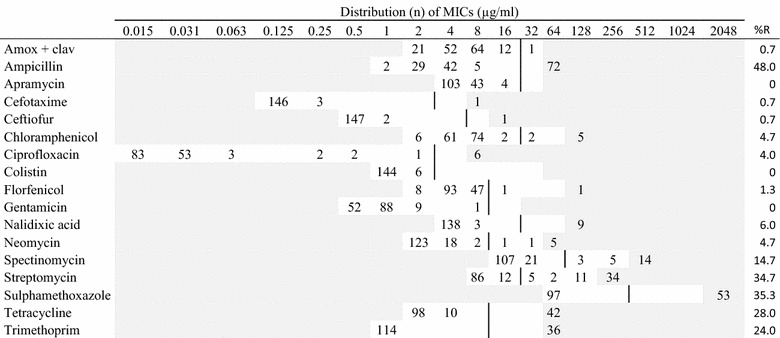
MIC distributions and occurrence of resistance of non-hemolytic *Escherichia coli* (n = 150) isolates from Danish mink (2014–2016)

Vertical lines indicate breakpoints for resistance (see breakpoint table in Additional file 1 A). White fields indicate test range for each antimicrobial. Values greater than the test range represent MIC values greater than the highest concentration in the range. MICs equal to or lower than the lowest concentration, are given as the lowest concentration in the test range

*R* resistance, *n* number of isolates, *amox* *+* *clav* amoxicillin with clavulanic acid (1:2)

**Table 3:**
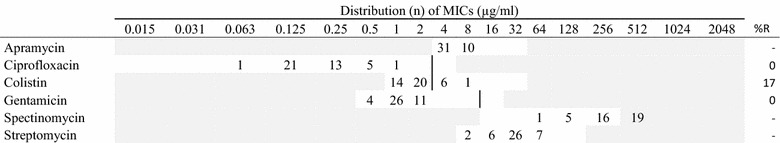
MIC distributions and occurrence of resistance of *Pseudomonas aeruginosa* (n = 41) isolates from Danish mink (2014–2016)

Vertical lines indicate breakpoints for resistance when available (see breakpoint table in Additional file 1 A). White fields indicate test range for each antimicrobial. Values greater than the test range represent MIC values greater than the highest concentration in the range. MICs equal to or lower than the lowest concentration, are given as the lowest concentration in the test range

*R* resistance, *n* number of isolates

**Table 4:**
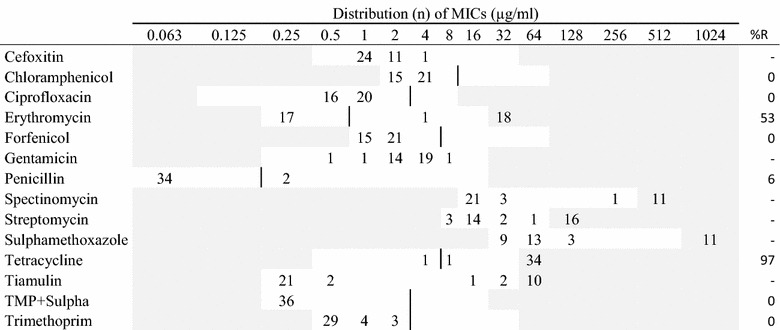
MIC distributions and occurrence of resistance of *Streptococcus canis* (n = 36) isolates from Danish mink (2014–2016)

Vertical lines indicate breakpoints for resistance when available (see breakpoint table in Additional file 1 B). White fields indicate test range for each antimicrobial. Values greater than the test range represent MIC values greater than the highest concentration in the range. MICs equal to or lower than the lowest concentration, are given as the lowest concentration in the test range

*R* resistance, *n* number of isolates, *TMP* *+* *Sulpha* trimethoprim with sulphamethoxazole (1:19)

**Table 5:**
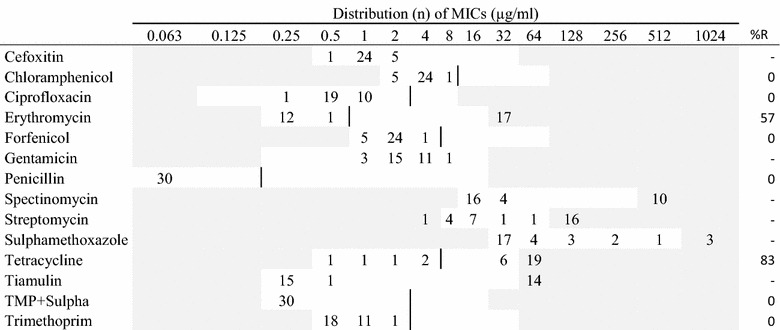
MIC distributions and occurrence of resistance of *Streptococcus dysgalactiae* (n = 30) isolates from Danish mink (2014–2016)

Vertical lines indicate breakpoints for resistance when available (see breakpoint table in Additional file 1 B). White fields indicate test range for each antimicrobial. Values greater than the test range represent MIC values greater than the highest concentration in the range. MICs equal to or lower than the lowest concentration, are given as the lowest concentration in the test range

**Table 6:**
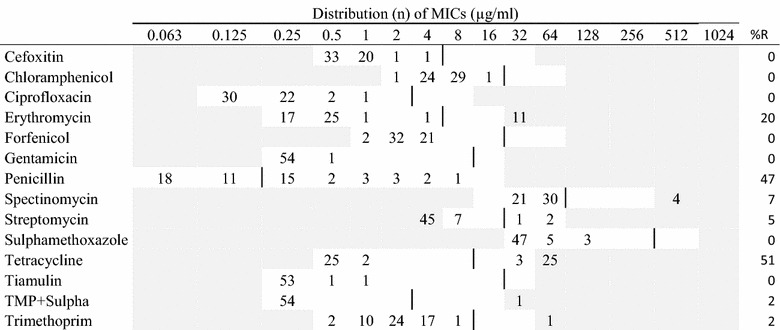
MIC distributions and occurrence of resistance of *Staphylococcus delphini* (n = 55) isolates from Danish mink (2014–2016)

Vertical lines indicate breakpoints for resistance (see breakpoint table in Additional file 1 B). White fields indicate test range for each antimicrobial. Values greater than the test range represent MIC values greater than the highest concentration in the range. MICs equal to or lower than the lowest concentration, are given as the lowest concentration in the test range

*R* resistance, *n* number of isolates, *TMP* *+* *Sulpha* trimethoprim with sulphamethoxazole (1:19)

**Table 7:**
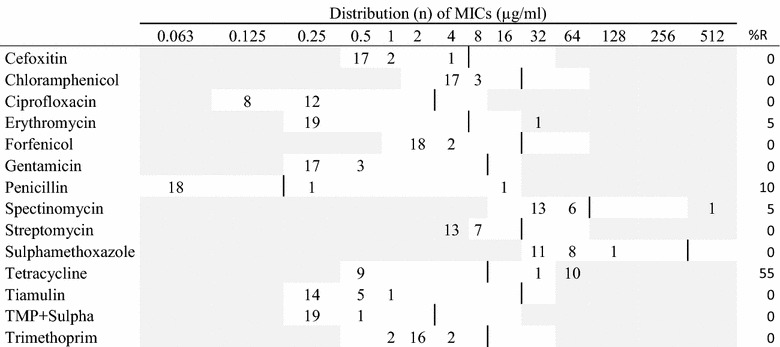
MIC distributions and occurrence of resistance of *Staphylococcus schleiferi* (n = 20) isolates from Danish mink (2014–2016)

Vertical lines indicate breakpoints for resistance (see breakpoint table in Additional file 1 B). White fields indicate test range for each antimicrobial. Values greater than the test range represent MIC values greater than the highest concentration in the range. MICs equal to or lower than the lowest concentration, are given as the lowest concentration in the test range

MIC values were interpreted using clinical breakpoints when available [see Additional file 1]. Since there are no approved breakpoints for mink pathogens, these interpretations must be regarded cautiously. Test ranges were as stated by Pedersen et al. [10]. Resistance percentages were calculated from isolates with MIC values above the breakpoint for resistance. In this study, the resistance level for each antimicrobial was considered low when <10% of the isolates were above the resistance breakpoint and considered high when resistance levels were >40%. Comparison between resistance levels in hemolytic and non-hemolytic *E. coli* was performed by using a Fisher’s exact test [11]. Results were considered significant when P < 0.05.

### Consumption of antimicrobial agents

Data on antimicrobial consumption in mink from 2007 to 2016 were extracted from the national veterinary prescription database, VetStat [12, 13]. VetStat data are considered to cover more than 99% of the total prescribed amounts of antimicrobials for veterinary use [14]. This study included all records on sales of antimicrobial drug for systemic use when (1) prescribed for mink, and/or (2) prescribed to mink farms with no other animal species recorded on the farm. The temporal developments in antimicrobial consumption were presented as annual kg active compound together with the trend in number of breeding females as a measure of population size.

To enable comparison of individual classes of antimicrobials, the consumption was measured in Defined Animal Doses. To adjust for fluctuations in population size, an estimated treatment proportion (TP) per year was calculated as;

$$TP = \mathop \sum \nolimits \frac{\text{active compound}}{{{\text{DADD}}\,{\text{kg}}\, *\,\left( {{\text{animal biomass}}\, *\,{\text{days}}} \right) }}$$

where DADDkg (mg/kg) is the number of defined daily dosage for treatment of one kg biomass, defined on product level as the recommended average daily dose, according to the principles described previously by Jensen et al. [5]; active compound was the annual antimicrobial use summarized on 4th or 5th ATCvet level [15]; the live animal biomass was estimated from number of breeding females registered at Kopenhagen Fur, and data on litter size and growth, as described by Jensen et al. [5]. A TP of 10 DADD/1000 biomass × days corresponds to 1% of the population biomass being treated on an average day.

## Results

### Resistance occurrence

In the hemolytic *E. coli* isolates, the highest occurrence of resistance was recorded for ampicillin (82.3%). Additionally, high resistance levels were found for streptomycin, sulphonamides, tetracyclines, and trimethoprim (>40%) (Table 1). For these compounds as well as spectinomycin, resistant isolates were recorded from any sampling site. For other tested antimicrobials, resistance levels were low.

Among the hemolytic *E. coli,* 45 different phenotypic resistance profiles were recorded. Only 19 of 158 isolates were sensitive to all 17 tested antimicrobials. Multiresistance, i.e. being resistant to three or more compounds, was recorded in 60% of all the isolates. The most common phenotypes were resistant to ampicillin-streptomycin-sulphonamide-tetracycline/trimethoprim (see Additional file 2). Mono-resistance was recorded in 10% of the isolates. Resistance for up to 10 compounds was recorded.

Resistance among the non-hemolytic *E. coli* isolates was also highest for ampicillin (48%), followed by streptomycin, sulphonamide, and trimethoprim (>25%) (Table 2). For these antimicrobials and tetracycline, resistant isolates were observed for all kind of samples. For other tested antimicrobials, resistance was at low levels.

The hemolytic and non-hemolytic *E. coli* isolates showed similar resistance patterns, e.g. both showed the highest level of resistance to ampicillin. However, higher levels of resistance were in general observed among the hemolytic isolates than among the non-hemolytic isolates (Tables 1, 2). The differences were statistically significant for ciprofloxacin (P < 0.03) and highly significant (P < 0.001) for ampicillin, streptomycin, sulphonamide, tetracycline and trimethoprim. Only for ciprofloxacin the resistance levels were higher in the non-hemolytic isolates (4%) than in the hemolytic isolates (1%) (Tables 1, 2).

All the 41 *P. aeruginosa* isolates were sensitive to ciprofloxacin and gentamicin. Colistin resistance was found in 17% of the isolates. All isolates were susceptible to apramycin in a concentration below 16 µg/mL (Table 3).

The two species of beta-hemolytic streptococci tested in this study, presented similar resistance patterns (Tables 4, 5). The majority of the 36 *S. canis* isolates and the 30 *S. dysgalactiae* isolates were resistant to tetracycline (97% and 83%, respectively). Additionally, high levels of resistance to erythromycin were found in both streptococci species with more than 40% of the isolates (Tables 4, 5). As all the isolates of *S. dysgalactiae* were sensitive to penicillin, and two of the *S. canis* isolates were resistant.

The two staphylococcus species tested in this study, presented similar resistance patterns except for penicillin (Tables 6, 7). Among the 55 *S. delphini* isolates the highest occurrence of resistance were found for tetracycline (51%), penicillin (47%) and erythromycin (20%) (Table 6). Among the 20 *S. schleiferi* isolates about half of the isolates were resistant to tetracyclines, but only two isolates were resistant penicillin (Table 7).

Only nine *S. aureus* isolates were available for testing. They were susceptible to the majority of the tested antimicrobials, while five of the isolates were resistant to penicillin and four to tetracyclines. One of the isolates was resistant to cefoxitin, suggesting that this *S. aureus* isolate was a methicillin-resistant *S. aureus* (MRSA).

### Antimicrobial consumption

**Fig. 1:**
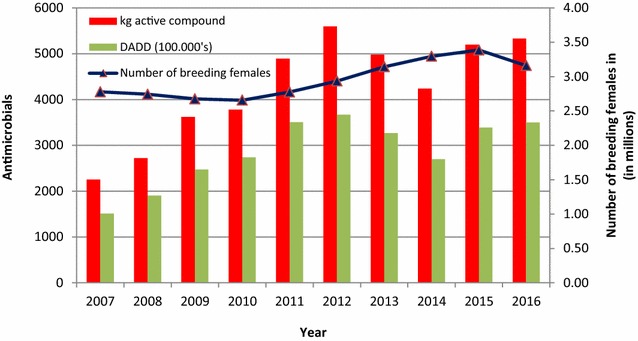
Antimicrobial prescriptions in Danish mink production (2007–2016). The prescription of antimicrobials given in kg active compound and DADD per year, and the curve indicating number of breeding females (in millions). DADD: defined animal daily dose is the assumed average maintenance dose needed to treat one kg animal

**Fig. 2:**
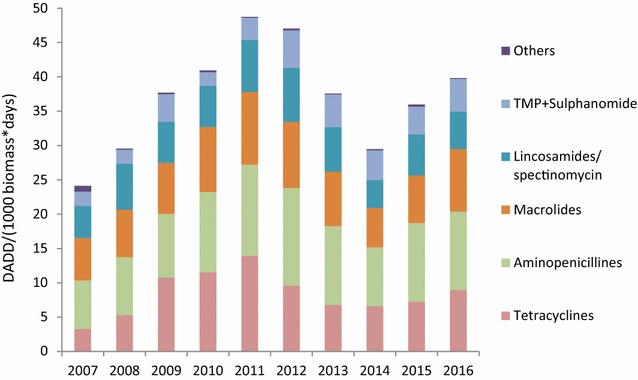
Antimicrobial prescriptions in the Danish mink production (2007–2016) by antimicrobial class. *DADD* defined animal daily dose is the assumed average maintenance dose needed to treat one kg animal. Others: Pleuromutilins, amphenicols, aminoglycosides, cephalosporins, colistin, fluoroquinolones, penicillin. TMP + sulphonamide: trimethoprim with sulphonamide

The overall antimicrobial consumption in the mink production measured in kg active compound, increased by 130% from 2007 to 2012, followed by a slight temporary decrease, most pronounced in 2014 (Fig. 1). From 2010 there has been an increase in number of breeding females, which may explain for some of the increase in usage (Fig. 1). Taking into account the changes in population size, the antimicrobial consumption increased by 109%, from 23 DADD/(1000 biomass × days) in 2007 to 48 DADD/(1000 biomass × days) in 2012 (Fig. 2). In 2014, the antimicrobial consumption decreased to around 30 DADD/(1000 biomass × days), and since increasing towards 40 DADD/(1000 biomass × days) in 2016. The rise during the period 2007–2012 was mainly related to the use of aminopenicillins (mainly amoxicillin), tetracyclines and macrolides, which are by far the most frequently used antimicrobials in the mink production (Fig. 2). Lincomycin in combination with spectinomycin has been commonly used, but it has been decreasing the past years. Cephalosporins and fluoroquinolones comprised less than 0.01% of the antimicrobial consumption in Danish mink during 2007–2012; amphenicols (florfenicol) comprised 0.06% and colistin comprised 0.2% of the consumption.

**Fig. 3:**
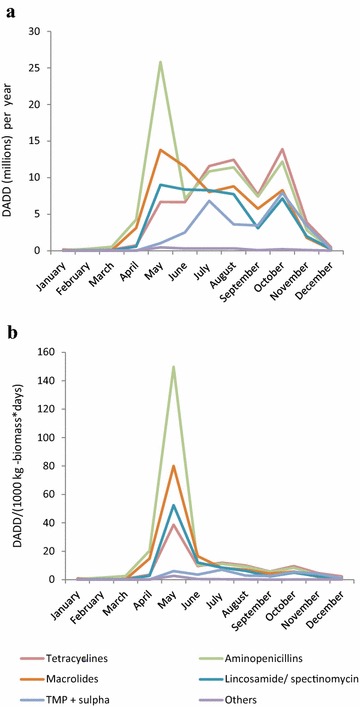
Seasonal patterns in antimicrobial prescriptions by antimicrobial class in the Danish mink production (2007–2016). **a** The graph is a monthly average from the time period 2007–2016, and illustrates the seasonal pattern in antimicrobial consumption. *DADD* defined animal daily dose is the assumed average maintenance dose needed to treat one kg animal. **b** The graph is a monthly average from the time period 2007–2016, and illustrates the seasonal pattern in antimicrobial consumption relative to the size of Danish mink production (monthly average, 2007–2016). DADD/(1000 kg – biomass * day) = number of DADD’s used within a given period per tonnes live biomass multiplied by number of days at risk within the time period (month), the unit describes the prescribed antimicrobials relative to the biomass on the farm, i.e. the decrease during autumn as the kits grow and the biomass increases. Others: Pleuromutilins, amphenicols, aminoglycosides, cephalosporins, colistin, fluoroquinolones, penicillin. TMP + sulpha: trimethoprim with sulphonamide

The seasonal pattern shows a dramatic peak in antimicrobial consumption in May (Fig. 3a). This is true for all antimicrobial classes, but most pronounced for the most used antimicrobials; aminopenicillins, macrolides, lincosamides with spectinomycin, and tetracyclines (Fig. 3a). The prescription of tetracycline also increases into the autumn (June–October), when the kits are growing and the biomass is significantly higher (Fig. 3b). In contrast, during the period from pelting (November–December) until the whelping season (May), the prescription of antimicrobial was very low (Fig. 3b).

## Discussion

In the present study, by far the highest level of resistance in *E. coli* was recorded for ampicillin, with 82.3% of the hemolytic and 48.0% of the non-hemolytic isolates. A similar observation was reflected in a previous study on antimicrobial susceptibility in mink pathogens, where the highest occurrence of resistance was found to ampicillin [2]. The same study showed that streptomycin, tetracyclines, sulphonamides, spectinomycin, and trimethoprim were associated with the highest levels of resistance [2]. These antimicrobial classes together with the aminopenicillins are also the most commonly used, but much fewer animals are treated with these drugs compared to aminopenicillins (Fig. 3b).

The resistance profiles of *E. coli,* with more than 50% of the isolates being resistant to sulphonamide and streptomycin, which are not commonly used in Danish mink, might be related to usage and/or to co-selection [16]. The potential of *E. coli* to transfer resistance plasmids and thereby spread antimicrobial resistance is well known; several resistance genes have been discovered, some genes give multiple resistances, and numerous resistance genes can be found within one isolate [17]. In this study, a high level of resistance to streptomycin was recorded, and as streptomycin is not used in mink, co-selection is the most likely cause [16, 17].

For both the hemolytic (1.9%) and non-hemolytic (0.7%) *E. coli*, a low number of cefotaxime resistant isolates were found. This resistance might indicate extended spectrum beta-lactamases (ESBL) status, but it was not investigated further in this study.

When comparing the hemolytic and non-hemolytic *E. coli*, resistance for most compounds was higher among the hemolytic isolates than among the non-hemolytic ones. A similar observation was made in a previous study, comparing hemolytic and non-hemolytic *E. coli* in Danish mink [18]. The reason for this is not known, and there is currently no evidence to suggest that these strains are more virulent to mink or more likely to be exposed to antimicrobials and subsequently develop resistance. However, this needs to be further investigated. In pigs, the hemolytic *E. coli* O149 is the most important pathogen in weaning diarrhea, and hemolysis is thought to be involved in the pathogenesis, although other toxins than hemolysin are known to be important [19].

In mink, *P. aeruginosa* is causative of hemorrhagic pneumonia, and this bacterium is well recognized because of its intrinsic resistance to most antimicrobials [8, 9]. High susceptibility was found to ciprofloxacin, colistin, and gentamicin. The few colistin-resistant strains found in this study might belong to the Gaussian distribution of the susceptible wild types (Table 3). In a previous study, all *P. aeruginosa* isolates were found susceptible to gentamicin and colistin [2].

In this study, both group G (*S. canis*) and group C (*S. dysgalactiae*) streptococci were investigated. In the two streptococcus species, high resistance levels to tetracycline were found; *S. canis*: 97% and *S. dysgalactiae*: 83%. High levels of resistance to tetracycline were also found in a previous study [2]. Resistance to macrolides, represented by erythromycin was high in data from 2008 [2] and this pattern was also found in the present study with more than 50% of the isolates being resistant in both species (Tables 4, 5). Whether the high levels of resistance to macrolides and tetracycline reflects the similarly high consumption of these compounds (Fig. 2) is uncertain. The tiamulin and spectinomycin MIC distributions showed a distinct division into two groups in both species. This might indicate the grouping of susceptible wild type and a resistant population (Tables 4, 5). Penicillin resistance was low in the streptococci despite high consumption of aminopenicillins; this is a pattern known also from other species, e.g. humans and cattle [20]. In this study, two *S. canis* isolates had a MIC value of 0.25 µg/mL to penicillin while the other isolates had MIC values ≤0.063 µg/mL. This needs to be further investigated.

The taxonomy of staphylococci has changed so that isolates from mink that were previously identified as *S. intermedius* are now considered to belong to the species *S. delphini*. Thus, the isolates reported by Pedersen et al. [2] as *S. intermedius* were likely all *S. delphini*. Among *S. delphini,* far the highest level of resistance was found to tetracycline (51%). A similar pattern was observed in 2008 [2], as high levels of resistant isolates were found to tetracycline, penicillin and erythromycin.

One of the *S. aureus* isolates was resistant to cefoxitin. This observation subsequently prompted an investigation of occurrence of MRSA in mink, and it has become evident that MRSA is widespread on Danish mink farms. The majority of the isolates are livestock-associated MRSA CC398, and belonging to spa-types t034 and t011, which are also most prevalent in pigs [21].

In general, the occurrence of resistance towards cephalosporins and fluoroquinolones is very low in bacterial isolates from Danish mink, most likely due to the very low consumption of the compounds both in Danish mink and other production animals in Denmark (Fig. 2) [20].

There was a marked increase in antimicrobial prescription in May (Fig. 3a). The reason is probably that that mink kits are born around early May, and the antimicrobials are mainly for treatment of pre–weaning mink diarrhea. In the peri-weaning period May–July, the prescription of aminopenicillins was 27% higher than macrolides and 75% higher compared to the use of tetracyclines. In contrast, tetracyclines were used 10% more than aminopenicillins and 65% more than macrolides in autumn. Thus aminopenicillins are in general used to treat pre- and post-weaning animals in the spring, whereas tetracyclines are used mainly in the almost full-grown animals in the autumn. Consequently, more animals can be treated with the given amount of aminopenicillins in the spring, than the tetracycline in the autumn. This explains the difference between Fig. 3a, b.

## Conclusions

For *E. coli*, high levels of resistance were recorded, especially among hemolytic isolates, to the most used compounds ampicillin and tetracyclines. High resistance levels to streptomycin and sulphonamides were recorded, probably due to co-resistance. The most commonly used antimicrobials are also reflected in the resistance patterns of Gram positive bacteria. The antimicrobial consumption data displays an overall decrease from 2011 to 2014, and then a gradual increase in 2015 and 2016.

There is a need for guidelines regarding treatment and susceptibility of relevant pathogens in Danish mink for veterinarians and farmers to optimize (and minimize) the use of antimicrobial compounds.

## Additional files

**Additional file 1.** Antimicrobial breakpoints (µg/mL). A) Breakpoint values for *Escherichia coli* and *Pseudomonas aeruginosa* applied in Tables 1, 2 and 3, B) Breakpoint values for *Staphylococcus* spp. and *Streptococcus* spp. applied in Tables 4, 5, 6 and 7.

**Additional file 2.** Resistance profiles recorded in the isolates of hemolytic *Escherichia coli* (n = 158) from Danish mink (2014–2016).
