# Supplementary material for: Antimicrobial resistance among pathogenic bacteria from mink (Neovison vison) in Denmark
Source: Acta Vet Scand. 2017 Sep 13;59:60. doi: 10.1186/s13028-017-0328-6 (PMC5598060; doi:10.1186/s13028-017-0328-6)
Supplement: Supplementary file 1 — Additional file 1. Antimicrobial breakpoints (µg/mL). A) Breakpoint values for Escherichia coli and Pseudomonas aeruginosa applied in Tables 1, 2 and 3, B) Breakpoint values for Staphylococcus spp. and Streptococcus spp. applied in Tables 4, 5, 6 and 7. [file 13028_2017_328_MOESM1_ESM.docx]

**Additional file 1. Antimicrobial breakpoints (µg/mL)**

A) Breakpoint values for *Escherichia coli* and *Pseudomonas aeruginosa* applied in Tables 1-3, B) Breakpoint values for *Staphylococcus* spp. and *Streptococcus* spp. applied in Tables 4-7.

**A**

| Antimicrobial | *E. coli* | | | Ref | *P. aeruginosa* | | | Ref |
| --- | --- | --- | --- | --- | --- | --- | --- | --- |
|  | S | I | R |  | S | I | R |  |
| Amoxicillin with clavulanic acid (1:2) | ≤8/4 | 16/8 | ≥32/16 | ^1^ | - | - | - | ^-^ |
| Ampicillin | ≤8 | 16 | ≥32 | ^1^ | - | - | - | ^-^ |
| Apramycin | ≤16 |  | ≥32 | ^5^ |  |  |  |  |
| Cefotaxime | ≤1 | 2 | ≥4 | ^1^ | - | - | - | ^-^ |
| Ceftiofur | ≤2 | 4 | ≥8 | ^2a^ | - | - | - | ^-^ |
| Chloramphenicol | ≤8 | 16 | ≥32 | ^1^ | - | - | - | ^-^ |
| Ciprofloxacin | ≤1 | 2 | ≥4 | ^1^ | ≤1 | 2 | ≥4 | ^1^ |
| Colistin | ≤2 |  | ≥4 | ^3^ | ≤2 |  | ≥4 | ^1^ |
| Florfenicol | ≤4 | 8 | ≥16 | ^2b^ | - | - | - | ^-^ |
| Gentamicin | ≤4 | 8 | ≥16 | ^1^ | ≤4 | 8 | ≥16 | ^1^ |
| Nalidixic acid | ≤16 |  | ≥32 | ^1^ | - | - | - | ^-^ |
| Neomycin |  |  | ≥16 | ^4^ | - | - | - | ^-^ |
| Spectinomycin |  |  | ≥128 | ^4^ |  |  |  |  |
| Streptomycin |  |  | ≥32 | ^4^ |  |  |  |  |
| Sulphamethoxazole | ≤256 |  | ≥512 | ^1^ | - | - | - | ^-^ |
| Tetracycline | ≤4 | 8 | ≥16 | ^1^ | - | - | - | ^-^ |
| Trimethoprim | ≤8 |  | ≥16 | ^1^ | - | - | - | ^-^ |

**B**

| Antimicrobial | *Staphylococcus* spp. | | | Ref | *Streptococcus* spp. | | | Ref |
| --- | --- | --- | --- | --- | --- | --- | --- | --- |
|  | S | I | R |  | S | I | R |  |
| Cefoxitin | ≤4 |  | ≥8 | ^1*^ |  |  |  |  |
| Chloramphenicol | ≤8 | 16 | ≥32 | ^1^ | ≤4 | 8 | ≥16 | ^1^ |
| Ciprofloxacin | ≤1 | 2 | ≥4 | ^1^ |  |  | ≥4 | ^4^ |
| Erythromycin | ≤0.5 | 1-4 | ≥8 | ^1^ | ≤0.25 | 0.5 | ≥1 | ^1^ |
| Florfenicol |  |  | ≥32 | ^4*^ | ≤2 | 4 | ≥8 | ^2b^ |
| Gentamicin | ≤4 | 8 | ≥16 | ^1^ | NA | NA | NA | ^3^ |
| Penicillin | ≤0.12 |  | ≥0.25 | ^1^ | ≤0.12 |  | ≥0.25 | ^1^ |
| Spectinomycin |  |  | ≥128 | ^4*^ |  |  |  |  |
| Streptomycin |  |  | ≥32 | ^4*^ |  |  |  |  |
| Sulphamethoxazole | ≤256 |  | ≥512 | ^1^ |  |  |  |  |
| Tetracycline | ≤4 | 8 | ≥16 | ^1^ | ≤2 | 4 | ≥8 | ^1^ |
| Tiamulin |  |  | ≥32 | ^4*^ |  |  |  |  |
| Trimethoprim with Sulphamethoxazole | ≤2/38 |  | ≥4/76 | ^1^ | ≤0.5/9.5 | 1/19 – 2/38 | ≥4/76 | ^2^ |
| Trimethoprim | ≤8 |  | ≥16 | ^1^ | ≤2 |  | ≥4 | ^3^ |

^1^ CLSI M100 S27:2017 [22], ^2^ CLSI VET01S ED3:2015 [23], ^2a^ CLSI VET01S ED3:2015 bovine [23], ^2b^ CLSI VET01S ED3:2015 porcine [23], ^3^ EUCAST (v 7.0 Breakpoint Tables) [24], ^4^ EUCAST Epidemiological cut-off values (ECOFFs) [25] ^5^Danmap 2015 bovine, porcine [16].

The presented values are human breakpoints unless other noted. S: susceptible, I: intermediate, R: resistant, Ref.: reference for breakpoint data. A) Antimicrobial breakpoints (µg/mL) for *E. coli* and *Pseudomonas aeruginosa.* Breakpoints for *E. coli* are adopted from Enterobactericeae. B) Antimicrobial breakpoints (µg/mL) for *Staphylococcus* spp. and *Streptococcus* spp. *Adopted from *Staphylococcus aureus*.
