# Supplementary material for: Antimicrobial resistance among pathogenic bacteria from mink (Neovison vison) in Denmark
Source: Acta Vet Scand. 2017 Sep 13;59:60. doi: 10.1186/s13028-017-0328-6 (PMC5598060; doi:10.1186/s13028-017-0328-6)
Supplement: Supplementary file 2 — Additional file 2. Resistance profiles recorded in the isolates of hemolytic Escherichia coli (n = 158) from Danish mink (2014–2016). [file 13028_2017_328_MOESM2_ESM.docx]

**Additional file 2. Resistance profiles recorded in the isolates of hemolytic *Escherichia coli* (n=158) from Danish mink (2014-2016).**

| Resistance profile^1^ | Number of isolates |
| --- | --- |
| AMP-TET | 23 |
| Sensitive | 19 |
| AMP-STR-SUL-TET | 14 |
| AMP-STR-SUL-TMP | 13 |
| AMP | 11 |
| AMP-STR-SUL-TET-TMP | 11 |
| AMP-STR-SUL | 6 |
| AMP-SPE-STR-SUL-TMP | 5 |
| AMP-SPE-STR-SUL-TET-TMP | 5 |
| AMP-TMP | 4 |

^1^ AMP = ampicillin, TET = tetracyclines, STR = streptomycin, SUL = sulphonamides, TMP = trimethoprim, SPE = spectinomycin

The table displays the ten most frequently occurring profiles of 45 different resistance profiles.
